# Cardiac biopsies reveal differences in transcriptomics between left and right ventricle in patients with or without diagnostic signs of heart failure

**DOI:** 10.1038/s41598-024-56025-1

**Published:** 2024-03-09

**Authors:** Christoffer Frisk, Sarbashis Das, Maria J. Eriksson, Anna Walentinsson, Matthias Corbascio, Camilla Hage, Chanchal Kumar, Mattias Ekström, Eva Maret, Hans Persson, Cecilia Linde, Bengt Persson

**Affiliations:** 1 Department of Cell and Molecular Biology, Science for Life Laboratory, Uppsala University, Box 596, 751 24 Uppsala, Sweden; 2 Department of Clinical Physiology, Karolinska University Hospital, 171 76 Stockholm, Sweden; 3 Department of Molecular Medicine and Surgery, Karolinska Institutet, 171 77 Stockholm, Sweden; 4 Translational Science and Experimental Medicine, Research and Early Development, Cardiovascular, Renal and Metabolism, BioPharmaceuticals R&D, AstraZeneca, 431 83 Gothenburg, Sweden; 5 Department of Thoracic Surgery, Karolinska University Hospital, 171 76 Stockholm, Sweden; 6 Department of Medicine, Karolinska Institutet, 171 77 Stockholm, Sweden; 7 Heart and Vascular Theme, Karolinska University Hospital, 171 76 Stockholm, Sweden; 8 Department of Medicine, Integrated Cardio Metabolic Center (ICMC), Karolinska Institutet, 141 57 Huddinge, Sweden; 9 Department of Clinical Sciences, Karolinska Institutet, Danderyd Hospital, 182 88 Stockholm, Sweden; 10 Department of Cardiology, Danderyd Hospital, 182 88 Stockholm, Sweden; 11 Department of Medical Biochemistry and Biophysics, Science for Life Laboratory, Karolinska Institutet, 171 77 Stockholm, Sweden

**Keywords:** Heart failure, Ischemic heart disease, Cardiac biopsy, Left ventricular dysfunction, Gene expression, Gene expression analysis, Computational biology and bioinformatics, Cardiology

## Abstract

New or mild heart failure (HF) is mainly caused by left ventricular dysfunction. We hypothesised that gene expression differ between the left (LV) and right ventricle (RV) and secondly by type of LV dysfunction. We compared gene expression through myocardial biopsies from LV and RV of patients undergoing elective coronary bypass surgery (CABG). Patients were categorised based on LV ejection fraction (EF), diastolic function and NT-proBNP into pEF (preserved; LVEF ≥ 45%), rEF (reduced; LVEF < 45%) or normal LV function. Principal component analysis of gene expression displayed two clusters corresponding to LV and RV. Up-regulated genes in LV included natriuretic peptides NPPA and NPPB, transcription factors/coactivators STAT4 and VGLL2, ion channel related HCN2 and LRRC38 associated with cardiac muscle contraction, cytoskeleton, and cellular component movement. Patients with pEF phenotype versus normal differed in gene expression predominantly in LV, supporting that diastolic dysfunction and structural changes reflect early LV disease in pEF. DKK2 was overexpressed in LV of HFpEF phenotype, potentially leading to lower expression levels of β-catenin, α-SMA (smooth muscle actin), and enhanced apoptosis, and could be a possible factor in the development of HFpEF. CXCL14 was down-regulated in both pEF and rEF, and may play a role to promote development of HF.

## Introduction

Heart failure (HF) affects 2–3% of the adult Western population^1^. Prevalence increases with age^2^ and is associated with considerable mortality and morbidity. In an aging population the proportion of HF with preserved left ventricular (LV) ejection fraction (EF) (HFpEF) is increasing^3^ and has in stable condition comparable prognosis^4^ to HF with reduced LVEF (HFrEF). But in contrast to HFrEF, there are few evidence-based, disease-modifying therapies in HFpEF^5^, which may be explained by different pathophysiology between these HF conditions. In patients undergoing CABG, LV dysfunction is very common and related to outcome^6^. It is thus important to identify mechanisms and genes involved in the transition from LV dysfunction to overt HF especially in patients with pEF who until recently lacked guidelines indicated HF medication.

While systemic neuroendocrine activation triggered by cardiomyocyte injury is key in HFrEF, more co-morbidities such as ischemia, hypertension and diabetes have been suggested as drivers of disease progression in HFpEF^7^. In experimental models incomplete relaxation of myocardial strips^8^ and increased passive cardiac stiffness by titin changes and increased interstitial fibrosis have been reported. In HFpEF patients this results in myocardial stiffness, LV hypertrophy and incomplete relaxation eventually leading to HF^7,9^.

At the molecular level, gene expression reflects the cell state, and changes in gene regulation reflect different diseases^10^. Therefore, studies of gene expression in heart tissue have a great potential to uncover the molecular mechanisms leading to HF, exploring differences in underlying pathophysiology. We have previously reported differences in gene expression in cardiac biopsies between patients with pEF and Normal LV systolic and diastolic function in an early report from the CABG-PREFERS study^11^ with a limited sample size.

Heart failure patients have altered LV myocardial structure and function and the right ventricle (RV) is often affected secondary to the LV dysfunction^12^. However, little is known regarding *gene expression* and potential differences between the LV and RV^13^. Myocardial biopsies obtained during coronary artery bypass grafting (CABG) provide a unique opportunity to study the gene expression in different LV function phenotypes in ischemic heart disease since patients undergoing CABG commonly have disturbances in LV function irrespective of HF symptoms^14^. Analysing the LV and comparing it to the RV is crucial as it underscores the distinct pathophysiological responses and adaptations of each chamber and helps us understand the differential impact of HF on the left and right sides of the heart^13^.

In this sub-study of CABG-PREFERS part of the PREFERS study programme we classified patients as described in our design paper^15^, based on LVEF, structural abnormality or diastolic dysfunction and NT-proBNP into those with 1. pEF phenotype (LVEF ≥ 45%), 2. rEF phenotype (LVEF < 45%), and 3. normal LV function. This is described more in detail in the “Methods” section.

We hypothesised that there are differences in gene expression and transcriptomics between LV and RV. As a secondary hypothesis-generating aim, we wanted to explore if there are any differences in gene expression in LV and RV between pEF and rEF patients and compared to Normal LV phenotype.

## Results

### Patients

A total of 29 patients were included of which nine (31%) with pEF phenotype, five (17%) with rEF phenotype, and 15 (51%) with normal LV phenotype. The patients’ clinical characteristics are summarised in Table 1. The majority were male, pEF patients were older (median age = 72 years) than rEF (median age = 67 years) and the normal phenotype group (median age = 65 years). pEF patients more commonly had a history of smoking, hypertension and worse renal function. Echocardiographic results for the three groups, classified as pEF, rEF and normal phenotype according to our study criteria^15^, are shown in Supplementary Table S1. A total of 42 biopsies were obtained—20 from LV and 22 from RV, whereof 14 patients with biopsies from both LV and RV.

**Table 1:** Patient characteristics.

Variable	pEF phenotype (n = 9)			rEF phenotype (n = 5)			Normal phenotype (n = 15)			p-value overall
	Median	Q1	Q3	Median	Q1	Q3	Median	Q1	Q3	
Age, years	72	69	77	67	65	70	65	63	70	0.06
Weight, kg	84	71	87	91	77	95	82	68	93	0.70
Height, cm	180	175	181	178	173	181	177	172	170	0.80
Body mass index, kg/m^2^	26	24	27	29	26	29	27	23	29	0.689

	n	%		n	%		n	%		
Sex (female) (n, %)	2	22		0	0		1	7		0.351
Smoking, previous (n; %)	6	66		2	40		8	53		0.627
Smoking, present (n; %)	0	0		0	0		0	0		
Previous medical history										
Atrial fibrillation	0	0		1	20		0	0		0.090
Hypertension	6	66		5	100		11	73		0.370
Diabetes	3	33		0	0		6	40		0.254
Peripheral vessel disease	1	11		0	0		0	0		0.329
Stroke/TIA	1	11		0	0		0	0		0.329
Previous percutaneous coronary intervention	1	11		1	13		2	13		0.899
HF status and physical findings										
NYHA I	3	33		1	20		5	33		
II	5	55		2	40		9	60		
III	0	0		2	40		1	6		
IV	0	0		0	0		0	0		

	Median	Q1	Q3	Median	Q1	Q3	Median	Q1	Q3	p-value overall
Systolic blood pressure, mmHg	165	143	171	149	145	159	140	131	148	0.093
Diastolic blood pressure, mmHg	84	75	88	86	83	92	80	79	83	0.322
Heart rate, beats/min	54	51	57	69	55	72	67	57	73	0.031
Laboratory										
eGFR ml/min/1.73 m^2^	60	0	92	75	59	88	89	71	99	0.266
Creatinine, µmol/l	84	63	102	96	81	114	79	73	93	0.530
NT-proBNP, ng/l	225	190	298	943	87	964	148	90	198	0.077
Potassium, mmol/l	3.8	3.7	3.9	4.3	3.8	4.3	4	3.8	4.3	0.221
Sodium, mmol/l	140	140	141	139	139	141	140	137	140	0.549
Uric acid, µmol/l	385	246	491	477	270	580	335	312	387	0.648
LDL, mmol/l	1.9	1.6	2	1.9	1.1	2.6	2.5	1.8	2.9	0.481
hsTroponin T, ng/l	14	5	17	18	13	20	9	5	11	0.038
HbA1c, mmol/mol	39	38	40	40	39	40	40	36	46	0.893
Glucose, mmol/l	5.7	5.5	6.5	7.2	6	8.3	7.8	6.9	9.4	0.034

Treatment	n	%		n	%		n	%		p-value overall
Antiplatelet	6	66		4	80		11	73		0.865
Anticoagulant	0	0		1	20		0	0		0.090
Nitrates	0	17		2	40		7	47		0.056
Beta blocker	5	55		4	80		8	53		0.574
ACE inhibitor	3	33		2	40		4	26		0.847
ARB	1	11		3	60		3	20		0.111
Statin	6	66		2	40		12	80		0.348

Data are expressed as median and quartiles (Q1;Q3) or number (%).

For NYHA, there is one missing value for the pEF phenotype group.

*ACE* angiotensin converting enzyme, *ARB* angiotensin II receptor blocker, *BMI* body mass index, *COPD* chronic obstructive pulmonary disease, *CABG* coronary artery bypass surgery, *NT-proBNP* N-terminal pro-brain natriuretic peptide, *TIA* transient ischemic attack.

As shown in Supplementary Table S1, pEF patients did not differ significantly from the Normal phenotype group for LVEF and LV volume index, while left atrial (LA) volume, LA-strain and E/eʹ ratio (indicating LV filling pressure) were significantly higher, and LV mass index (LVMI) borderline increased consistent with pEF. RV diameter and longitudinal RV function were not different between the three phenotypes. More details are given in the Methods section.

### Differences in gene expression between LV and RV

**Figure 1:**
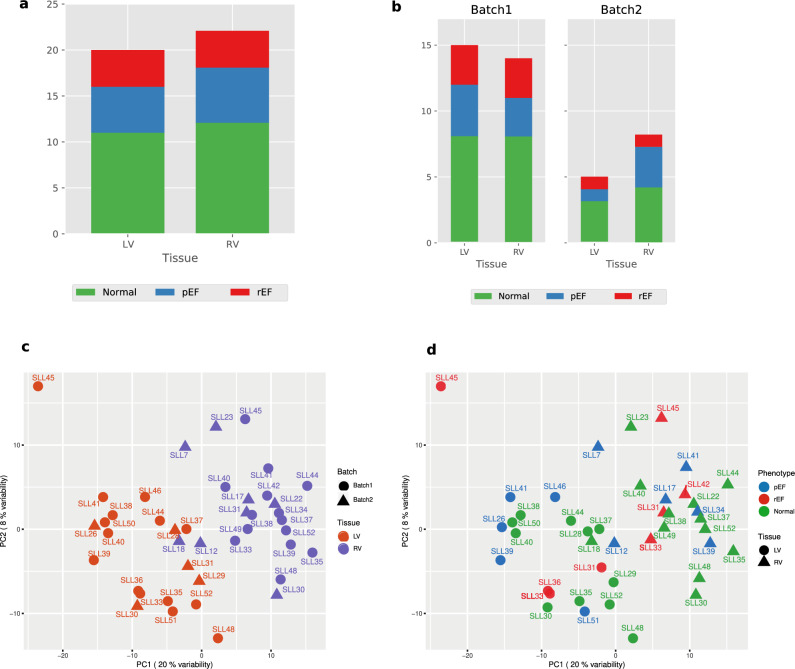
Gene expression profile discriminates left and right ventricles. (**a**) Number of tissue samples and conditions from left and right ventricles. (**b**) Distribution of tissue samples in sequencing batches. (**c**) Principal component analysis (PCA) score plot with the two principal components (PC1 and PC2) plotted on the x- and y-axis, respectively. Each data point represents one sample, which is color-coded according to the tissue and shaped according to the sequencing batch. (**d**) Same plot as shown in “c” but colour-coded based on conditions and shape of the points indicates tissue.

The distribution in gene expression between pEF, rEF and Normal groups is shown in Fig. 1a with Normal being the largest group and rEF the smallest. RNA expression was analysed in two batches as visualised in Fig. 1b. In the principal component analysis (PCA plot in Fig. 1c) two clusters were identified, one with LV samples and one with RV samples, reflecting differences in gene expression between ventricular tissue types. A similar PCA plot with the different LV dysfunction and Normal phenotypes (Fig. 1d) shows no difference between the groups, indicating that ventricular tissue differences were more pronounced than differences between LV dysfunction types.

### Differential gene expression between LV and RV

**Figure 2:**
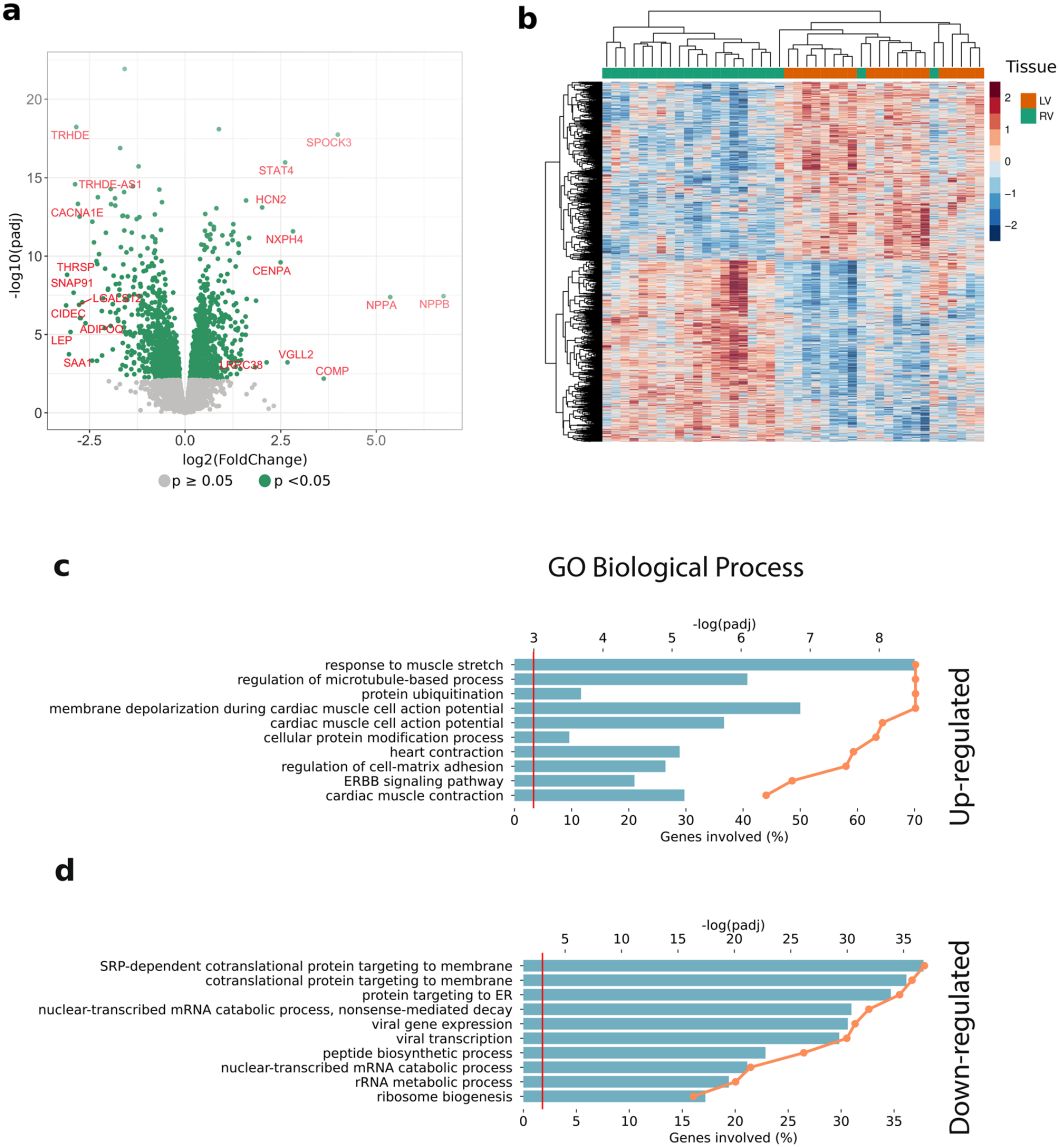
Differential expression and functional analysis between left ventricle (LV) and right ventricle (RV) tissues. (**a**) Volcano plot of the differentially expressed genes. The x-axis represents fold change in log2 scale of LV versus RV while the y-axis indicates the p-values in − log10 scale. Each point represents a gene, and significantly expressed genes are highlighted in green. Top 10 significantly differentially expressed (FDR-adjusted p-value < 0.05) genes each from up and down regulated are labelled with gene symbols. (**b**) Heatmap showing normalised expression values of the differentially expressed genes in all the tissue samples and clustered using unsupervised hierarchical clustering. Rows and columns indicate tissue samples and differentially expressed genes respectively. The expression values are standardised sample-wise for each gene. Gene ontology (GO) annotation of the differentially expressed genes in term of biological process. (**c**) Up-regulated and (**d**) Down-regulated genes. The horizontal bars show percentage of the genes with the corresponding GO annotations (scale at bottom x-axis), The orange lines represent significance of the corresponding GO annotations (scale at top x-axis) as calculated by Enrichr.

A total of 2383 differently expressed genes (DEGs) were found. The *volcano plot* in Fig. 2a shows the top 10 significantly differently expressed up- and down-regulated genes in RV versus LV. The up-regulated genes in LV versus RV included natriuretic peptides NPPA and NPPB, transcription factors/coactivators STAT4 and VGLL2, ion channel related HCN2 and LRRC38. Among the down-regulated genes in LV versus RV were the adipose tissue hormone LEP, galectin-related protein LGALS12, lipid droplet protein CIDEC, thyroid hormone-responsive protein THRSP, calcium channel subunit CACNA1E, acute phase protein SAA1, and the adipocyte-specific protein ADIPOQ. The *heat-map* in Fig. 2b of the differently expressed genes shows a separation between RV samples to the left (green) and LV samples to the right (orange), except two RV samples that intermixed with the LV samples. The general pattern of down-regulated genes in RV matching up-regulated genes in LV and reverse is clearly visible.

*Functional analysis* using Enrichr^16^ showed that the up-regulated genes in LV versus RV are associated with cardiac muscle contraction, cytoskeleton, cellular component movement (Fig. 2c), while the genes down-regulated in LV are associated with SRP (signal recognition particle) dependent co-translational protein targeting to membrane and protein targeting to the endoplasmic reticulum (Fig. 2d).

### Upstream regulators LV versus RV

We analysed upstream regulators using Ingenuity Pathway Analysis (IPA) on the 2383 DEGs between LV and RV. With Fishers Exact test p-values ≤ 0.05 a total of 1791 upstream regulators were found connected to the DEGs.

**Figure 3:**
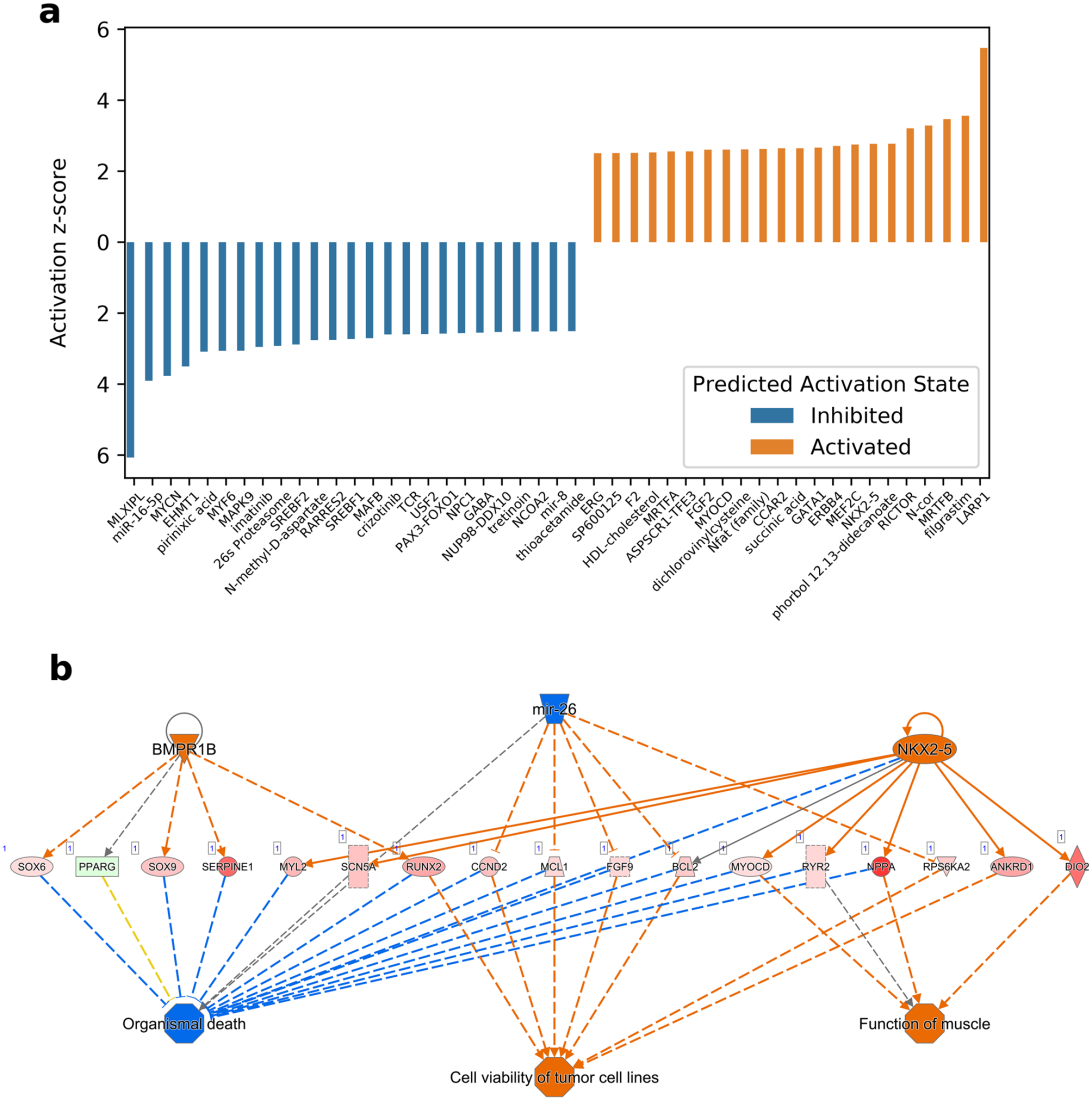
Functional analysis and predicted regulatory effect of the differentially expressed genes between LV and RV. (**a**) Shows analysis of upstream regulators using Ingenuity Pathway Analysis (IPA) analysis on the 2383 DEGs. The bar plot shows 47 with activation z-score of ≥  + 2.5 or ≤  − 2.5, i.e. 25 predicted to be inhibited and 22 predicted activated. (**b**) Shows the network with the highest consistency score, having a predicted effect of activating muscle function, cell viability of tumour cell lines and organismal death. This network has 3 regulators (BMP31B, mir-26 and NKX2-5) operating on 17 DEGs including NPPA, RYR2 and DIO2.

A total of 47 regulators with activation scores ≥  + 2.5 and ≤ –2.5 were predicted to be activated or inhibited, respectively (Fig. 3a). The regulators with the highest activity scores were LARP1, MRTFB, MEF2C, NKX2-5 and GATA1, and the regulators with the lowest activity scores were MLX1PL, mir-16-5p and MYCN. Predicted regulatory networks based on the same DEGs counted up to 31. The network with the highest consistency score showed a predicted activation of cell viability of tumour cell lines and muscle function, and inhibition of organismal death. The network consists of the regulators BMPR1B, mir-26 and NKX2-5 operating on 17 DEGs including NPPA, DIO2 and RYR2. (Fig. 3b).

### Tissue-wise comparison of pEF, rEF and normal phenotypes

**Figure 4:**
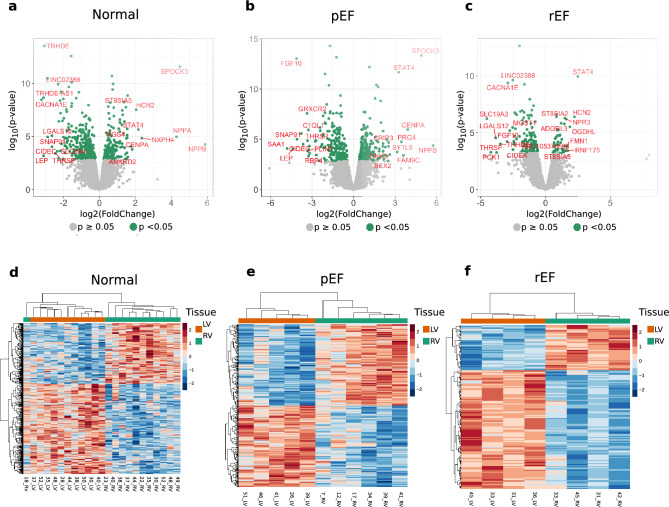
Differential expression analysis between left ventricle (LV) and right ventricle (RV) tissues in different conditions. Volcano plots showing fold change distribution of the differentially expressed genes in different HF phenotypes. The x-axis represents fold change in log2 scale of LV versus RV while the y-axis indicates the p-values in − log10 scale. Each point represents a gene, and significantly expressed genes are highlighted in green. Top 10 significantly differentially expressed (FDR-adjusted p-value < 0.05) genes each from up and down regulated are labelled with gene symbols. HF phenotypes: (**a**) normal, (**b**) preserved ejection fraction (pEF), (**c**) reduced ejection fraction (rEF). Heatmaps showing normalised expression values of the differentially expressed genes in all the tissue samples and clustered using unsupervised hierarchical clustering. Rows and columns indicate tissue samples and differentially expressed genes respectively. The expression values are standardised sample-wise for each gene. HF phenotypes: (**d**) Normal, (**e**) pEF, (**f**) rEF.

In Fig. 4a–c, results are shown from tissue-wise comparison between LV and RV of gene expression in Normal, pEF and rEF, respectively. The top 10 up- and down-regulated genes in each condition are indicated with gene symbols in red. Some genes were up- or down-regulated under multiple phenotypes, while others only appeared in one phenotype, as detailed below.

The heat-maps (Fig. 4d–f) show the differently expressed genes for Normal, pEF and rEF. The LV samples group together, and the RV samples group together, separately, emphasising that there were major differences in gene expression between these two tissue types. Only among the Normal, one sample (18_RV) clustered with the LV samples, but at the borderline, as indicated by the tree structure at the top.

In the LV samples, we found significant differences in gene expression between pEF and Normal, as previously reported^11^ but not between rEF and Normal. In the RV samples, there were no significant differences neither between pEF and Normal nor between rEF and Normal. The genes exhibiting differential expression in the LV samples between pEF and Normal are detailed in Supplementary Table S2.

### Up- and down-regulated genes in LV versus RV with regard to phenotype

**Figure 5:**
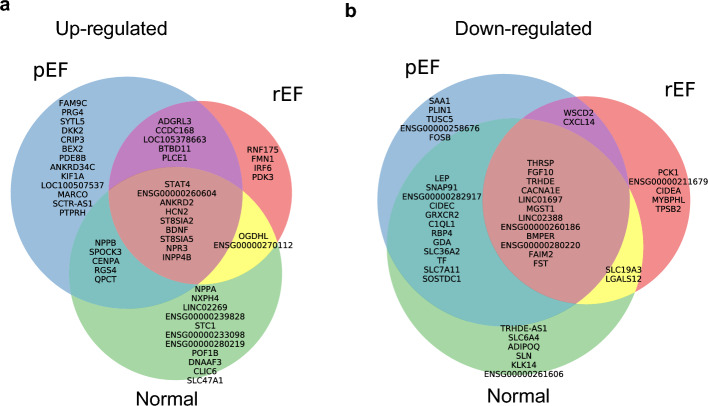
Venn-diagram of differentially expressed genes in different HF phenotypes. Top 20 differentially expressed genes filtering on log_2_ fold change 1 in each HF phenotype. (**a,b**) Show up- and down-regulated, respectively. Genes are displayed in descending order of log_2_ fold change.

All significantly up- or down-regulated genes for pEF, rEF and Normal are listed in Supplementary Tables S3 and S4 and briefly summarised below. The top 20 up- and down-regulated genes, respectively, in LV versus RV in each of the three phenotypes are shown in Fig. 5. Genes that are up- or down-regulated with a log fold change of at least 1 in other HF phenotypes(s) are listed at the appropriate place in the Venn diagram of Fig. 5. In this group of genes, nine are upregulated in all three phenotypes.

### Patterns of functional characteristics of differently expressed genes

**Figure 6:**
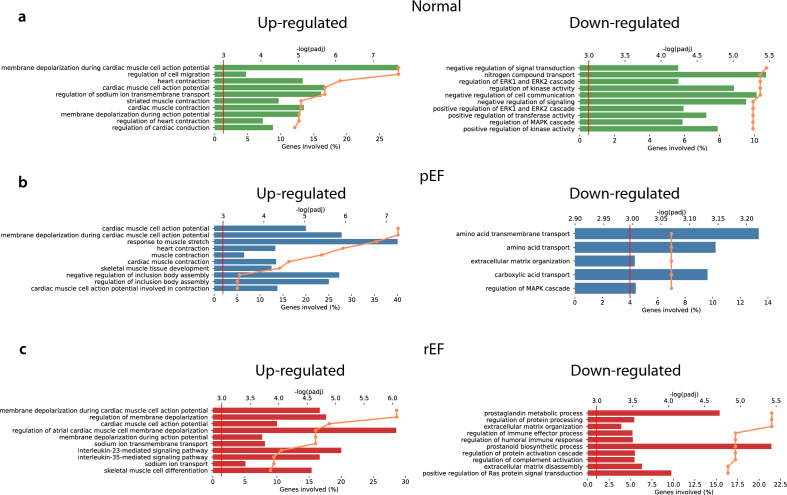
Functional classification of the differentially expressed genes in different HF phenotypes. (**a**) Shows GO annotations of biological process of up and down-regulated genes in LV versus RV in Normal. The horizontal bars display percentage of the genes with the corresponding GO annotations (scale at bottom x-axis), the orange lines represent significance of the corresponding GO annotations (scale at top x-axis) as calculated by Enrichr. (**b**) Displays pEF and (**c**) rEF.

The functional properties of differently expressed genes in Normal, pEF and rEF are illustrated in Fig. 6. In patients with normal LV function, the most significant biological functions among up-regulated genes in LV involved membrane depolarisation during cardiac muscle cell action potential, regulation of cell migration, heart contraction, and cardiac muscle cell action potential. Down-regulated genes were involved in negative regulation of signal transduction, regulation of ERK1 and ERK2 cascade and nitrogen compound transport.

In patients with pEF phenotype, up-regulated genes in LV were involved in cardiac muscle cell action potential and membrane depolarisation during cardiac muscle cell action potential, including the calcium channel CACNA2D1, sodium pump ATP1A2, potassium/sodium channel HCN2, and sodium channel SCN2B. In response to muscle stretch, we found dystrophin (DMD), ryanodine receptor RYR2, and cardiac LIM protein CSRP3 reflecting increased muscle stretch, and cardiac muscle contraction. Down-regulated genes in LV for pEF showed association with amino acid- and carboxylic acid transport but also extracellular matrix organisation.

For the groups rEF and pEF, we found up-regulation of genes in LV with biological functions of membrane depolarisation during cardiac muscle cell action potential (HCN2, SCN5A and SCN2B).

### Upstream regulators in pEF

**Figure 7:**
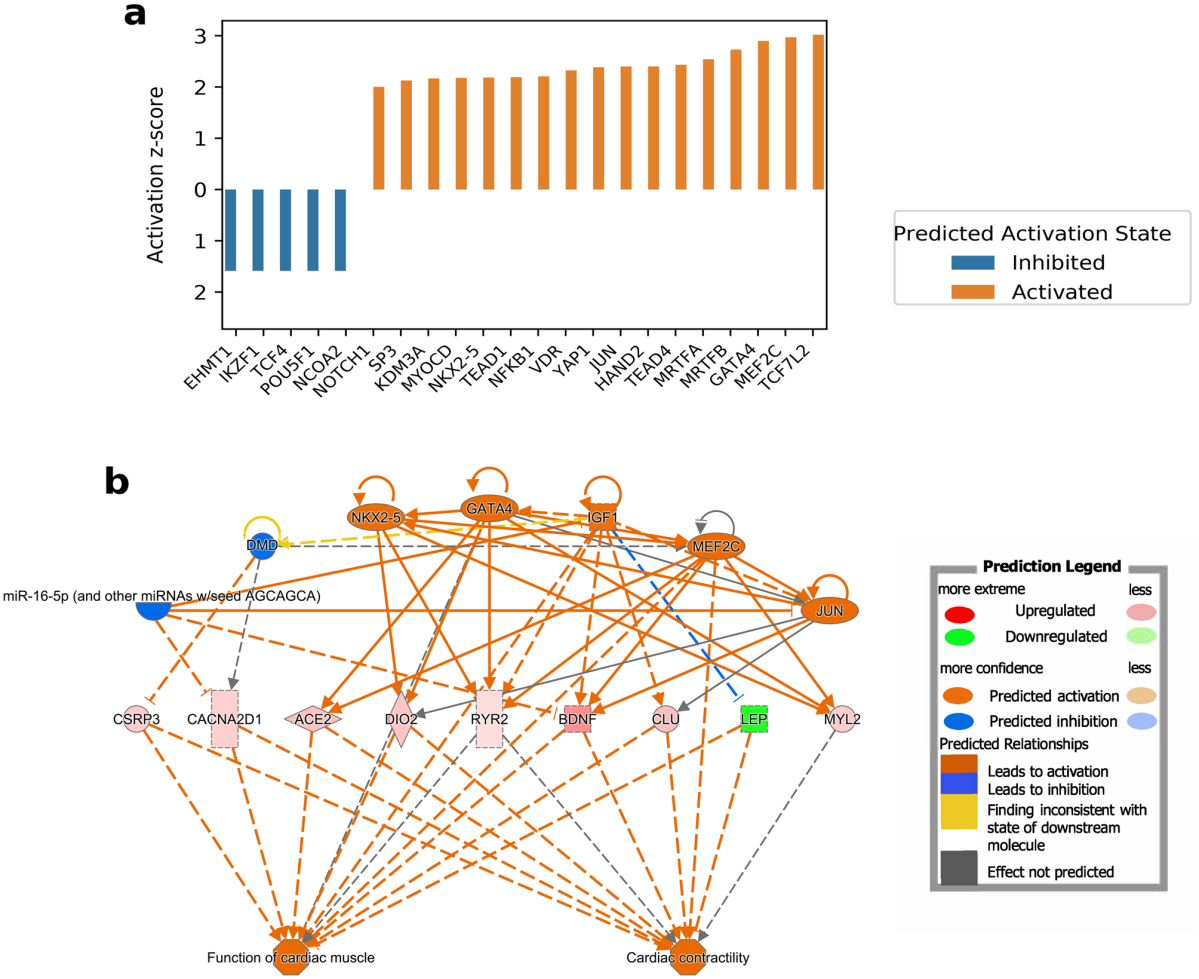
Predicted upstream regulator and regulatory effect network of the differentially expressed genes between LV and RV in pEF. (**a**) Shows upstream regulators predicted utilising the Ingenuity Pathways Analysis (IPA). Out of the upstream regulators 22 have an activation score above + 2 or below − 2. In (**b**), calculated activation z-scores are shown for 11 functional groups based upon enrichment of 96 DEGs involved in cardiovascular system development and functionally separated into 10 networks with significant p-values ≤ 0.05. (**c**) Shows a network with a predicted activation of cardiac muscle function and cardiac contractility through the upstream regulators CSRP3, CACNA2D1, ACE2, DIO2, BDNF and CLU.

IPA analysis revealed four regulatory networks and 1013 upstream regulators with 22 having an activation score above + 2 or below − 2 (Fig. 7a). The regulatory effect network with the highest consistency score showed predicted activation of cardiac contractility and cardiac muscle function through NKX2.5, GATA4, IGF1 MEF2C and JUN effects on the up-regulated BDNF, DIO2, ACE2, RYR2 and CLU, and down-regulated LEP (Fig. 7b).

## Discussion

In our study of patients with stable symptomatic coronary artery disease, we found clear differences in gene expression primarily between LV and RV but also in LV dysfunction phenotypes. Thirdly, we found differential LV gene expression primarily between the pEF and Normal ventricular function phenotypes respectively, supporting that early disturbances in diastolic function also entail LV disease.

### Patient material

The distribution of phenotypes in our study largely corresponds to a National Swedish registry study of patients that had undergone CABG^14^. In the registry most (65%) had normal LVEF defined as LVEF > 50% and 24% had reduced LVEF defined as LVEF < 50%. Only a minority had overt heart failure: 2.9% had HFpEF and 8.2% had HFrEF^14^. In our study, most patients (51%) had normal LV function, 9 (31%) had pEF, and 5 (17%) had rEF phenotypes. This means that LV dysfunction without clinical HF is common in CABG patients and that the distribution of our study patients was representative for CABG patients in Sweden.

### Differential gene expression between LV and RV

The top 10 up-regulated genes in LV versus RV (Fig. 2a) include those coding for the natriuretic peptides NPPA and NPPB. STAT4 was observed as an upstream regulator in our previous study^11^ on RNA expression in pEF versus Normal, and has been shown to be important in both innate and adaptive immune responses^17,18^. HCN2 coding for potassium/sodium hyperpolarisation-activated cyclic nucleotide-gated channel 2 contributes to the native pacemaker currents in the heart^19^. CENPA (centromere protein A) has been reported to be essential for cardiac progenitor cell proliferation^20^. VGLL2 (transcription cofactor vestigial-like protein 2) has been suggested to be involved in the programming of slow muscle fibres^21^. The expression of CENPA and VGLL2 might reflect the distinct ways in which the LV and RV respond to and manage cardiac stress, and their capacity to undergo remodelling. LRRC38 codes for a leucine-rich repeat-containing protein that potentially functions as auxiliary subunits of BK potassium channels^22^. Three genes—SPOCK3, NXPH4 and COMP—have so far not been associated with heart tissue or diseases.

Among other genes up-regulated in LV versus RV were ST8SIA2, which codes for alpha-2,8-sialyltransferase 8B, modulating cardiomyocyte excitability^23^; NPR3, which codes for natriuretic peptide receptor 3, suggested as a clearance receptor for NPPA, NPPB and NPPC^24^; and ADGRL3, which codes for adhesion G protein-coupled receptor L3, which plays a role in cell–cell adhesion^24^.

The top 10 down-regulated genes in LV versus RV (Fig. 2a) include several genes that are involved in lipid metabolism; LGALS12, THRSP, CIDEC, ADIPOQ, LEP, and SAA1. A down-regulation of these genes suggests an altered metabolic profile that likely is a result of the functional differences between the ventricles and also points to a lower usage of lipid metabolism, as energy source, in LV.

The functional analysis showed that up-regulated genes in LV were associated with cardiac muscle contraction, cytoskeleton, cellular component movement (Fig. 2c), while the genes down-regulated in LV were associated with protein synthesis, and negative regulation of signalling (Fig. 2d). The latter might be a relative effect of no major groups of genes being down-regulated in LV.

### Physiological importance of differences

The differences between LV and RV are distinct beginning with their different embryological origin, size, shape and gross morphology. Further, LV and RV differ in composition and orientation of myocardial fibres, function, and coronary circulation^12,25^. In terms of physiological differences LV and RV operate within different arterial pressure ranges, where RV is facing a much lower afterload from pulmonary circulation characterised by low resistance and high compliance, in contrast to the high afterload imposed on LV. This results in morphological adaptations such as thinner RV wall thickness and lower mass, lower wall stress and consequently lower oxygen demand compared to LV^25^. Response to pressure overload results in RV dilatation and later decrease in systolic function while in LV the diastolic function is affected early^12,26^.

In comparing LV and RV under all conditions, MEF2C and NKX2-5 emerged as predicted to be key regulators (see Fig. 3a). MEF2C, a transcription activator, is crucial in cardiac morphogenesis and vascular development. Studies involving MEF2C knockout mice have shown its significant impact in decreasing cardiac contractility^27^. MEF2C, along with GATA4 and NKX2-5, also enhances the expression of MYL2 (myosin light chain 2)^28^, a protein vital for cardiac development, contractility, and overall cardiac function.

IPA revealed the network with the highest consistency score included predicted inhibited noncoding microRNA miR-26 through its activation of muscle function and organismal death (Fig. 3b). miR-26 has proven an important regulator in hypoxia linking it to mitochondrial protection and reduced apoptosis^29^. Down-regulation of miR-26 has been observed to protect cardiomyocytes from hypoxia induced apoptosis in rat cardiomyocyte cultures^30^. It is possible that this observation in LV is a protective measure in response to hypoxia not present in RV. But microRNAs may be increased in HF and serve as drug targets. One such example is CDR132L, a first-in-class miR-132 inhibitor, which was found to attenuate and even reverse HF in preclinical models, that was also tested in humans with dilated cardiomyopathy and elevated miR-132 in plasma. In a recent early human study^31^ of patients who like in our study had chronic ischemic heart disease but with HFrEF, CDR132L treatment resulted in sustained miR-132 reduction in plasma, reduction in NT-proBNP and QRS narrowing indicative of reverse fibrosis. The significance of down-regulations of microRNAs found in our study is unclear but it is likely that microRNAs may serve as future drug-targets to potentially halter the transition from pEF to HFpEF.

### Tissue-wise comparison between LV and RV according to phenotypes

We observed phenotype-related differences in DE genes. Normal and pEF phenotype had more DE genes and larger log2FC values (Fig. 4). In both cases the previously mentioned NPPB and SPOCK3 obtained the highest log2FC. Comparing DE genes between phenotypes showed an overall high overlap of both up- and down-regulated genes between Normal and pEF (Fig. 5). We recognise that the differences between the LV and RV were greater than that between LV function phenotypes. But we are interested in early stages of LV dysfunction in the transition to overt HF. This is relevant since not only HF at the time of CABG but also diastolic LV dysfunction^6^ is associated with worse outcome. Many patients undergoing CABG may have signs of LV dysfunction that are overlooked due to lack of HF symptoms. We focus on the implications of such subclinical changes with the ultimate goals to find treatable pathways to avoid transition into overt HF and in particular for pEF patients in whom little therapy exists. Therefore, we believe it is of interest to report the differences in gene expression related to phenotype.

*Up-regulated genes in LV versus RV in both LV dysfunction phenotypes but not in normal* include ADGRL3 (Adhesion G Protein-Coupled Receptor L3) reported to be more expressed in dilated cardiomyopathy patients than in normal heart donors^32^. Expression levels of PLCE1 (Phospholipase C Epsilon 1) in myocardial ischaemia–reperfusion infused rat hearts increases over time in hypoxia and overexpression has been seen to promote inflammation through the NF-kB signalling pathway^33^. In humans, PLCE1 has been reported to be overexpressed during HF^34^, suggesting that PLCE1 contributes to the inflammatory process and subsequent possible impact on heart tissue remodelling and function.

*Up-regulated genes in LV versus RV in pEF only* include proteoglycan 4 (PRG4) which has been shown to be up-regulated in calcified regions of aortic valves^35^ and might have a similar role in the HFpEF heart; and DKK2, coding for dickkopf 2, which is expressed in the developing heart^36^. DKK2 expression has been reported to result in lower expression levels of β-catenin, α-SMA (smooth muscle actin), and enhanced apoptosis^37^. For KIF1A (Kinesin Family Member 1A) it has been shown that overexpression in the Drosophila heart causes reduced contractility and valve remodelling^38^, potentially contributing to heart disease. Also STRIT1/DWORF (LOC100507537), which codes for sarcoplasmic/endoplasmic reticulum calcium ATPase regulator DWORF, is up-regulated. DWORF enhances sarcoplasmic reticulum Ca^2+^ uptake and myocyte contractility. In mouse models of rEF, DWORF overexpression has been shown to restore cardiac function^39^. A similar function might be possible in pEF. Improved calcium handling is the mechanism of action of an implantable cardiac device to treat heart failure “cardiac contractility modulation” resulting in improved systolic and diastolic function^40^. We found improvements in quality of life and diastolic function by cardiac contractility modulation HFpEF in a recent pilot study^41^.

*Up-regulated genes in LV versus RV in rEF only* include FMN1, coding for formin 1, which plays a role in the formation of adherens junction and the polymerisation of linear actin cables^24^, vital for maintaining cellular structure and integrity. FMN1 has previously been reported to be up-regulated in HF and suggested to lead to the activation of RhoA/ROCK signalling pathways, which are implicated in the further development of cardiac hypertrophy^42^. Pyruvate dehydrogenase (PDH) lipoamide kinase isozyme 3, PDK3, is a part of the PDH complex playing a critical role in the mitochondrial energy production. While PDK3 has not been directly connected with HF, reduced PDK4 expression levels have been observed in human failing hearts^43^, possibly indicating that a compensatory mechanism might lead to up-regulation of PDK3.

*Up-regulated genes in LV versus RV in pEF and normal phenotypes but not in rEF* include centromere protein A (CENPA), which has been shown to be essential for cardiac progenitor cell proliferation^20^; NPPB, which together with NPPA, is known to be up-regulated in ventricular myocardium during cardiac stress^44^; and G-protein signalling regulator RGS4, which in humans has been observed as up-regulated in failing myocardium^45^. RGS4 inhibits G-protein-mediated signalling resulting in cardiomyocyte hypertrophy^46^.

*Up-regulated genes in LV versus RV in all three HF phenotypes* include the previously mentioned STAT4, which has been reported as overexpressed in hypoxia-exposed human ovarian cancer tissue^47^, and ANKRD2 (Ankyrin repeat domain-containing protein 2), which functions as a negative regulator of myocyte differentiation and may interact with both sarcoplasmic structural proteins and nuclear proteins to regulate gene expression during muscle development and in response to muscle stress^24^. Natriuretic Peptide Receptor 3, NPR3, is a receptor that binds to the natriuretic peptide NPPA which is important in the regulation of blood pressure. Natriuretic peptides (NP) have a cardioprotective effect and augmented effects of NPs have been reported through transgenic Osteocrin in mouse models. Osteocrin has been found to competitively bind to NPR3 on ANP cultured cells, suggesting that its cardioprotective effect is due to the inhibition of NPR3-dependent NP^48^.

*Down-regulated genes in LV versus RV in pEF and rEF but not in normal* include CXCL14 (coding for C-X-C Motif Chemokine Ligand 14). CXCL14 exerts a synergistic effect with IRX1 to attenuate myocardial fibrosis and cardiomyocyte apoptosis^49^. Inhibition of CXCL14 expression has been postulated to promote heart failure, leading to increased fibrosis formation and dramatically impaired cardiac function^49^. CXCL14 has also been shown to be inhibited by miR-1278 in tissue subject of myocardial infarction in mouse model^50^. CXCL14 is a target of miR-1278, which exerts a protective role in cardiac tissue against myocardial infarction.

*Down-regulated genes in LV versus RV in pEF only* SAA1 (serum amyloid A1) has been identified as a hub gene in a protein–protein interaction network associated with left ventricle stress cardiomyopathy in human test patients^51^. It plays a significant role in mitigating cardiac remodelling by inhibiting inflammatory pathways such as NF-κB/p38/JNK and TGFβ/Smad^52^. The down-regulation of SAA1 in pEF may contribute to adverse cardiac remodelling.

*Down-regulated genes in LV versus RV in rEF only* include CIDEA (Cell Death-Inducing DFFA-Like Effector A) known to activate apoptosis^53^ and myosin-binding protein-H like (MYBPHL), of which loss in a mouse model has been shown to impair the LV function^54^.

*Down-regulated genes in LV versus RV in pEF and Normal but not in rEF* include retinol binding protein 4 (RBP4), which can induce cardiomyocyte hypertrophy in mouse^55^, and Cell death-inducing DFFA-like effector C (CIDEC) which is proposed to be a trigger for diabetic cardiomyopathy (DCM) through its involvement in insulin resistance. Gene silencing of CIDEC shows reduced inflammation, cardiac dysfunction and myocardial hypertrophy in DCM rat models^56^.

ENSG00000282917, which is anti-sense to CORIN^57^—a serine-type endopeptidase involved in NPPA and NPPB processing^58,59^. Overexpression of CORIN in mice with dilated cardiac myopathy has showed improved cardiac contractility and reduced cardiac fibrosis^60^ so a decreased expression of the inhibiting ENSG00000282917 suggests improvement of these functions.

Complement C1q like 1 (C1QL1) has been shown to stimulate angiogenesis of endothelial cells and has been proposed as a therapeutic target for recovery of ischemic heart disease^61^.

*Down-regulated genes in all three phenotypes* include FGF10 (Fibroblast Growth Factor 10), which has been proposed as a relevant target for heart regeneration due to its cardiomyocyte renewal and fibrosis inhibition^62^, and BMPER (BMP Binding Endothelial Regulator), for which deficiency in mice has shown decreased cardiomyocyte size and increased cardiac vessel density and thus likely to have a role in regulation of these characteristics^63^. The interpretation of this finding must be seen in the light of all patients in our study having coronary artery disease.

### Patterns of functional characteristics of differentially expressed genes

When we compared gene expression in LV, there were clearly visible differences between pEF and Normal, as previously reported^11^. However, when we compared gene expression in RV between the different conditions, there were no significant differences between pEF and Normal or between rEF and Normal. These findings may be related to the LV dysfunction in an early phase of HF, where only LV is affected.

GO analysis of up-regulated genes showed cardiac muscle cell action potential in all phenotypes (Fig. 6). Changes in action potential and K^+^ channel expression prolongation has been suggested as playing a role in the development and progression of heart disease^64^. Only Normal and pEF showed up-regulation of cardiac muscle contraction indicating a lower activity in rEF, which also possessed down-regulation of acute inflammatory response. This suggests that the inflammatory response, as a defensive mechanism to protect injured tissue, has reached a stagnant state. Both pEF and rEF express a down-regulation of extracellular matrix (ECM) organisation indicating a disruption in cardiac homeostasis, a likely result from pressure overload^65^.

### Upstream regulators and effect networks in pEF

Conclusive results could only be derived from the analysis of the pEF patients (Fig. 7). The regulator with the lowest activation z-score and a predicted inhibiting state is mir-16-5p. The function of mir-16-5p is still unclear. However, knockout of mir-16-5p leads to both increased cell viability and angiogenesis. mir-16-5p has therefore been proposed as a new therapeutic target in myocardial infarction^66^. Euchromatic Histone Lysine Methyltransferase 1 (EHMT1) is predicted as an inhibited transcription regulator. Inactivation of EGHMT1 has been observed to promote pathological hypertrophy in mouse models^67^.

The previously mentioned regulatory transcription factors, in LV-RV all phenotypes, NKX2-5 and MEF2C are also found in HFpEF specifically (Fig. 7).

Myocardin Related Transcription Factor A and B (MRTFA, MRTFB) are among the most activated upstream regulators, together with the coactivator serum response factor (SRF). They are involved in controlling myogenic differentiation^68^ and cytoskeleton regulation during development^69^. SRF, through MRTF, enables the heart to respond to skeletal stress through the balance of monomers and filaments^70^.

The most activated upstream regulator was Transcription factor 7 like 2, TCF7L2 (Fig. 7a), associated with impaired glucagon-like peptide-1 (GLP-1) signalling and a strong regulator of insulin production^71^ with genetic variants associated with increased risk of type 2 diabetes^72^. TCF7L2 has an important role in WNT signalling, and genetic variants of TCF7L2 have been connected with reduced insulin secretion^73^ and increased cardio-sympathetic activity. Imbalance in sympathetic/parasympathetic activity is associated with cardiovascular mortality^74^ and TCF7L2 has therefore been proposed as a potential cardiovascular risk factor^73^.

### Clinical relevance

Our findings are restricted to patients with coronary artery disease. We can therefore not quantify to which extent ischemic heart disease or the distinct LV function phenotypes explain our findings. The strength of this study is in the revealed gene expression differences between LV and RV found in myocardial biopsies.

Finally, gene editing and gene silencing are becoming a reality in HF treatment in view of the potential to develop antisense oligonucleotides that prevent the translation of mRNA into target proteins. Additionally, the CRISPR technique may allow gene editing in the treatment of or prevention of HF in the future. Therefore, exploratory studies such as our identifying gene expression differences in early stages of HF are of importance for the development of future therapies.

### Limitations and strengths

However, there are some limitations in the study, such as relatively small number of patients used and unequal distribution between the pEF, rEF and Normal groups. We had very few women in our study and mostly in the pEF group. This is a limitation especially in view of the proportion of women with overt HF and pEF (HFpEF) in clinical practise.

In order to obtain myocardial biopsies, we have chosen patients undergoing elective CABG enabling us to safely obtain tissue samples. To identify the pEF group, we used echocardiographic evaluation according to current international guidelines^2^. We could confirm that a proportion of patients indeed had pEF characteristics. Due to limited amounts of biopsy material, we had to use the entire biopsy to get deeper RNA sequencing for better sensitivity. Consequently, we were unable to perform any validation experiments. A larger number of patients can add more insight into differences in gene expression between these two groups.

### Conclusions

In our myocardial biopsy study of patients undergoing elective CABG with different LV function phenotypes (pEF, rEF and normal) gene expression differed between LV and RV, which is compatible with the different physiological roles of the two ventricles. Secondly, we suggest that differences between LV and RV may be due to most patients having no diagnosed HF and thus RV dysfunction is rarely present in early LV dysfunction. In patients with pEF compared to Normal LV function, differences in gene expression were predominantly observed in LV suggesting that early stages of diastolic dysfunction also involve primarily the left ventricle. Our results call for further studies.

## Methods

### Patients

Patients enrolled were scheduled for elective CABG without concomitant valve surgery. They all had angina pectoris with or without a previous myocardial infarction. Cardiac biopsies were obtained during CABG for analysis of mRNA expression in the myocardial tissue. All patients were assessed at a baseline visit 4–8 weeks prior to CABG by clinical characteristics, echocardiography and blood sampling including natriuretic peptides. From the ongoing study CABG-PREFERS^15^ we now report data from the initial patients.

*Descriptive data* are presented as median and quartiles (Q1;Q3) or number (%), and comparisons between groups were performed by Kruskal–Wallis and chi-square tests as appropriate.

### Definitions

Preserved LVEF was defined at the time of study design as LVEF ≥ 45%^15^. The patients were divided into three groups according to echocardiography, NT-proBNP concentration and HF guidelines definitions^2^ given below. The group with echocardiographic characteristics and increased NT-proBNP concentration indicative of HFpEF^2,15^ was called *pEF phenotype* for the purpose of this study and was used as a representative for HFpEF even when not showing signs or symptoms of HF. Patients with LVEF < 45% were called *rEF phenotype*, while the *Normal phenotype* group had LVEF ≥ 45%, no echocardiographic signs of HFpEF^75^ or elevated NT-proBNP concentration*.*

Based on echocardiographic findings and NT-proBNP results patients were classified into 3 phenotype groups: pEF, rEF and Normal physiology, in a stepwise manner using established criteria^76,77^ and an established analysis method^78^, without regard to signs or symptoms of HF. In the *pEF group* EF ≥ 45% was required together with a majority of the following 7 abnormal criteria: TVI septal eʹ < 7 (cm/s) or TVI lateral eʹ < 10 (cm/s), TVI mean of septal and lateral eʹ < 9 (cm/s), E/eʹ > 8, TR velocity ≥ 2.8 m/s, LA volume index > 34 mL/m^2^, and NT-proBNP (pg/ml) ≥ 125 pg/ml. If one of the above criteria was lacking, at least 1 of the following was required: LVMI female > 95 g/m^2^ or LVMI male > 115 g/m^2^, RWT ≥ 0.42 and decrease in E/A during Valsalva by > 50%. The *rEF group* was defined by EF < 45% and the *Normal group* defined by EF ≥ 45% and absence of the above criteria for pEF. In equivocal cases, classification was performed by consensus of two experts (M.J.E. and H.P., blinded for clinical characteristics) in line with previous experiences from the CHARM substudy^78^.

### Echocardiography

Transthoracic Doppler echocardiography was performed according to guidelines as previously reported^15^. A Vivid 9 ultrasound system (Vingmed-General Electric, Horten, Norway) was used in all studies. Images were digitally stored on a dedicated server, and data analysis was performed offline on the EchoPAC workstation (GE EchoPAC sw only, Norway) by one experienced sonographer. The mean value of 3 cardiac cycles was calculated for each variable.

### Tissue collection

From patients undergoing CABG, core needle biopsies were taken from the lateral wall of the left and right ventricles before initiation of cardiac arrest and stored in − 70 °C as previously described^15^ and used for mRNA analysis. Patients were prepared for surgery according to standard clinical routines with placement of a central venous line in the internal jugular vein, an arterial line in the distal radial artery and a peripheral venous line in the brachial vein. A midline sternotomy was performed and one or two mammary arteries were procured for usage as conduits^79^.

### RNA extraction and sequencing

Total RNA was extracted using the RNeasy Fibrous Tissue Mini Kit (#74704, Qiagen). RNA libraries for sequencing were prepared using poly-A selection and the Illumina RNA strand-specific TruSeq Stranded mRNA Sample prep kit with 96 dual indexes (Illumina, CA, USA) according to the manufacturer’s instructions with the following changes. The protocols were automated using an Agilent NGS workstation (Agilent, CA, USA) using purification steps as described^80,81^.

Clustering was done by ‘cBot’ and samples were sequenced on HiSeq2500 (HiSeq Control Software 2.2.58/RTA 1.18.64) with a 2 × 125/2 × 150 setup using ‘HiSeq SBS Kit v4’ chemistry. The Bcl to FastQ conversion was performed using bcl2fastq-v2.17.1.14 from the CASAVA software suite. The quality scale used was Sanger/phred33/Illumina 1.8+.

### Analysis of transcriptome (RNA-Seq) data

Whole transcriptome sequencing was performed for each biopsy. Initial quality checking of the sequencing raw reads was performed to identify potential outliers before doing further analysis using FastQC. Sequencing paired-end reads were mapped towards the human reference genome (version GR38) using Star Aligner^82^, and Ensembl genome annotation (version 37) was used for subsequent analysis. Reads that mapped to the exons of the coding genes were counted using HTSeq^83^. Genes with count values of zero (i.e. no read detected) in all samples were filtered out before further analysis. Before normalisation, lowly expressed genes were filtered out using proportion test per condition and multiple testing correction as per NOIseq manual^84^. Normalisation of the count data was performed using the Trim mean of M-values (TMM) approach^85^.

*Batch correction* of the data and differential gene expression analysis (false discovery rate (FDR) < 0.05) was performed using the NOISeq package^84^. Analysis and plots were generated in R using ggplot2.

*Principal component analysis* (PCA) was performed on the log_2_ transformed values of normalised batch corrected expression values using “princomp” function in R.

Clustering analysis of the differentially expressed genes (DEGs) was performed using unsupervised hierarchical clustering. Normalised expression data were standardised before plotting as heatmap using pheatmap package for R environment.

### Functional analysis of the dysregulated genes

Gene set enrichment analysis for Gene Ontology (GO) terms with focus on biological process (BP) and cellular component (CC) was performed for the DEGs (using gene symbols as input) using Enrichr^16^ with the probability density function as p-value model. The enrichment was tested using Fisher’s exact test with corrected p-value < 0.05. The DEGs were further analysed through the use of IPA^86^ (Ingenuity Pathways Analysis; QIAGEN Inc., https://www.qiagenbioinformatics.com/products/ingenuity-pathway-analysis). This tool uses the information in the Ingenuity® Knowledge Base to assess signalling and metabolic pathways, identify potential upstream regulators, and explore regulatory effect networks, as well as disease and biological functions that are likely to be perturbed based on a data set of interest (in our case, the DEGs). The IPA upstream analysis^86^ was performed to predict which regulators (i.e. any gene, protein or miRNA) that are activated or inhibited based on a calculated Activation Z-score, to explain the observed DEG changes in pEF phenotype group vs. Normal phenotype group. The IPA regulatory effect network analysis generated hypotheses for how a phenotype, function or disease is regulated by activated or inhibited upstream regulators. In our study, regulatory effect network analysis was used to specifically study the impact of the identified upstream transcription factor regulators on downstream heart disease functions, given the observed gene expression changes in pEF phenotype group vs. Normal phenotype group.

### Ethics statement

This study was conducted according to the Declaration of Helsinki and approved by the Regional Ethics Committee Stockholm (EPN; Dnr 2013/1869-31/1). All patients were included following oral and written informed consent.

### Supplementary Information

Supplementary Information.

Supplementary Table S2.

Supplementary Table S3.

Supplementary Table S4.

Supplementary Figure S1.

## Data Availability

RNA-seq data have been deposited in the EMBL-EBI ArrayExpress database (www.ebi.ac.uk/arrayexpress) under accession number E-MTAB-13553.
