# Supplementary material for: Cardiac biopsies reveal differences in transcriptomics between left and right ventricle in patients with or without diagnostic signs of heart failure
Source: Sci Rep. 2024 Mar 9;14:5811. doi: 10.1038/s41598-024-56025-1 (PMC10924960; doi:10.1038/s41598-024-56025-1)
Supplement: Supplementary file 1 — Supplementary Information. [file 41598_2024_56025_MOESM1_ESM.docx]

Supplementary Material to:

**Cardiac biopsies reveal differences in transcriptomics between left and right ventricle in patients with or without diagnostic signs of heart failure**

Christoffer Frisk^1^, Sarbashis Das^1^, Maria J Eriksson^2,3^, Anna Walentinsson^4^, Matthias Corbascio^3,5^, Camilla Hage^6,7^, Chanchal Kumar^4,8^, Mattias Ekström^9,10^, Eva Maret^2,3^, Hans Persson^9,10^, Cecilia Linde^6,7^ and Bengt Persson^1,11^

^1^Department of Cell and Molecular Biology, Science for Life Laboratory, Uppsala University, S-751 24 Uppsala, Sweden; ^2^Karolinska University Hospital, Department of Clinical Physiology, S-171 76 Stockholm, Sweden; ^3^Karolinska Institutet, Department of Molecular Medicine and Surgery, S-171 77 Stockholm, Sweden;^4^Translational Science and Experimental Medicine, Research and Early Development, Cardiovascular, Renal and Metabolism, BioPharmaceuticals R&D, AstraZeneca , S-431 83 Gothenburg, Sweden; ^5^Karolinska University Hospital, Department of Thoracic Surgery, S-171 76 Stockholm, Sweden; ^6^Karolinska Institutet, Department of Medicine, S-171 77 Stockholm, Sweden; ^7^Karolinska University Hospital, Heart and Vascular Theme, S-171 76 Stockholm, Sweden; ^8^Integrated Cardio Metabolic Center (ICMC), Department of Medicine, Karolinska Institutet, S-141 57 Huddinge, Sweden; ^9^Karolinska Institutet, Department of Clinical Sciences, Danderyd Hospital, S-182 88 Stockholm, Sweden; ^10^Danderyd Hospital, Department of Cardiology, S-182 88 Stockholm, Sweden; ^11^Department of Medical Biochemistry and Biophysics, Science for Life Laboratory, Karolinska Institutet, S-17177 Stockholm, Sweden

**Supplementary Table 1:** Echocardiographic measures in three patient groups, classified as pEF, Normal or rEF phenotypes.

|  | pEF (n=9) | | | Normal (n=15) | | | rEF (n=5) | | |  |  |  |  |
| --- | --- | --- | --- | --- | --- | --- | --- | --- | --- | --- | --- | --- | --- |
| Variable | Median | Q1 | Q3 | Median | Q1 | Q3 | Median | Q1 | Q3 | p-value overall | p-value pEF vs rEF | p-value  pEF vs Normal | p-value  Normal vs rEF |
| ***Left ventricular indices*** |  |  |  |  |  |  |  |  |  |  |  |  |  |
| LV e-d diameter (mm) | 47 | 46 | 52 | 48 | 45 | 50 | 56 | 54 | 56 | <0.012 | 0.021 | 0.57 | 0.003 |
| Interventricular septum e-d thickness (mm) | 12.0 | 11.0 | 14.0 | 11.0 | 10.0 | 12.5 | 14.0 | 14.0 | 14.0 | 0.099 |  |  |  |
| LV posterior wall e-d thickness (mm) | 10 | 9 | 10 | 9 | 8 | 9 | 10 | 9 | 11 | 0.167 |  |  |  |
| Relative wall thickness | 0.42 | 0.33 | 0.43 | 0.37 | 0.34 | 0.40 | 0.38 | 0.32 | 0.39 | 0.701 |  |  |  |
| LV e-d volume (ml) | 120 | 116 | 146 | 113 | 96 | 125 | 162 | 147 | 180 | 0.031 | 0.099 | 0.304 | 0.009 |
| LV e-d volume index (ml/m2) | 67 | 57 | 71 | 55 | 49 | 67 | 75 | 63 | 102 | 0.056 |  |  |  |
| LV mass index (g/m^2^) | 107 | 86 | 113 | 84 | 78 | 102 | 133 | 129 | 140 | 0.002 | 0.04 | 0.117 | 0.000 |
| LV ejection fraction (%) | 56 | 54 | 58 | 59 | 55 | 61 | 39 | 37 | 44 | 0.001 | 0.015 | 0.189 | 0.000 |
| LV global longitudinal strain (absolute values, %) | 15.8 | 15.8 | 16.3 | 18.3 | 16.9 | 19.7 | 11.1 | 11.0 | 14.0 | 0.002 | 0.046 | 0.110 | 0.001 |
| ***Right ventricular indices*** |  |  |  |  |  |  |  |  |  |  |  |  |  |
| RV e-d diameter (mm) | 38 | 34 | 40 | 36 | 35 | 38 | 41 | 35 | 48 | 0.258 |  |  |  |
| TAPSE (mm) | 21.2 | 21.0 | 22.9 | 21.4 | 18.8 | 24.1 | 21.0 | 17.7 | 21.3 | 0.371 |  |  |  |
| RV area change (%) | 0.47 | 0.40 | 0.48 | 0.50 | 0.48 | 0.53 | 0.36 | 0.34 | 0.50 | 0.032 | 0.874 | 0.017 | 0.076 |
| RV strain (absolute values %) | 20.0 | 18.9 | 21.9 | 18.1 | 16.6 | 20.0 | 14.9 | 11.5 | 14.9 | 0.008 | 0.002 | 0.350 | 0.012 |
| Inferior vena cava diameter (mm) | 15.2 | 10.9 | 17.9 | 13.2 | 10.6 | 15.1 | 15.7 | 12.6 | 17.6 | 0.289 |  |  |  |
| ***Left and right atrial indices*** |  |  |  |  |  |  |  |  |  |  |  |  |  |
| LA volume index (mL/m^2^) | 37.7 | 35.8 | 40.3 | 32.0 | 27.6 | 34.7 | 37.8 | 35.8 | 42.4 | 0.007 | 0.985 | 0.006 | 0.023 |
| LA volume index >34 (ml/m^2^) (n;%) | 9 | 100 |  | 4 | 27 |  | 4 | 80 |  | <0.05 |  |  |  |
| LA strain global (%) | 23.9 | 23.0 | 26.0 | 29.5 | 25.5 | 33.0 | 15.0 | 13.6 | 25.2 | 0.010 | 0.726 | 0.013 | 0.016 |
| RA area (cm^2^) | 17.0 | 16.3 | 19.2 | 15.0 | 13.5 | 16.3 | 17.8 | 15.9 | 20.3 | 0.049 | 0.844 | 0.039 | 0.058 |
| RA strain global (%) | 30.6 | 27.5 | 35.8 | 34.8 | 32.3 | 37.4 | 32.2 | 12.2 | 39.0 | 0.617 |  |  |  |
| ***Diastolic function variables*** |  |  |  |  |  |  |  |  |  |  |  |  |  |
| E/e´ | 10.9 | 9.10 | 11.5 | 7.58 | 6.81 | 8.81 | 9.18 | 7.33 | 9.46 | 0.013 | 0.181 | 0.003 | 0.339 |
| E/A ratio | 0.9 | 0.7 | 1.1 | 0.8 | 0.8 | 1.0 | 1.2 | 1.0 | 1.3 | 0.620 |  |  |  |
| Mitral deceleration time (ms) | 253 | 195 | 284 | 231 | 205 | 256 | 236 | 233 | 246 | 0.547 |  |  |  |

Values are median, Q (quartile) or n (number), % (percentage). LV = left ventricular; e-d = end-diastolic; e-s = end-systolic; RV = right ventricular, TAPSE = tricuspid annular plane systolic excursion; LA = left atrial; E = mitral inflow Doppler E-wave velocity; A = mitral inflow Doppler velocity at atrial contraction; E/A ratio = a ratio of mitral E to A velocity; e’ = early diastolic mitral annular tissue Doppler velocity; E/ e’ ratio – a ratio of mitral E to mean value of septal and lateral tissue e’ velocity. C

### Comments to Supplementary Table 1

The Supplementary Table 1 shows echocardiographic results in the study population. pEF patients did not differ significantly from the Normal phenotype group for LVEF and LV volume index, while left atrial (LA) volume, LA-strain and E/e’ ratio (indicating LV filling pressure) were significantly higher, and LV mass index (LVMI) borderline increased consistent with pEF. In spite of per definition normal LVEF in the pEF group these patients had reduced global longitudinal LV strain (GLS) compared to Normal group indicating reduced systolic LV function. In the rEF group, LVEF was indeed low with a median of 39%, while LV diameter (median 56 mm) and LV volume (median 162 ml) were enlarged in comparison to pEF and Normal. Furthermore, LV GLS was significantly lower in rEF in comparison to both pEF and Normal (median 11.1% compared to 15.8% for pEF and 18.3% for Normal). RV diameter and longitudinal RV function were not different between the three phenotypes. When RV systolic function was assessed using RV strain rEF patients showed significant lower systolic RV function compared to pEF and Normal group.

**Supplementary Table S2:** Excel file. Differentially expressed genes in LV samples between pEF and Normal, represented by logFC (log fold change) and logCPM (log counts per million). Also LR (likelihood ratio) and Pvalue (adjusted p-value) are given.

**Supplementary Table S3.** Excel file. Comparative Gene Expression between Left Ventricle (LV) and Right Ventricle (RV) across All, Normal, rEF, and pEF conditions. Values are presented as LogFC (log2FC), LR (likelihood ratio) and Pvalue (adjusted p-value).

**Supplementary Table S4:** Excel file. Comprehensive table of all differentially expressed (DE) genes between LV and RV, represented by logFC and logCPM (log counts per million) across all conditions. Columns are gradient-coloured, with blue indicating positive values and red indicating negative values. Genes with a significant p-value < 0.05 are marked with an "x."

**Supplementary Figure S1.** Distribution of RNA-seq Read Mapping for Each Patient. The bar chart represents the categorization of RNA-seq reads for every patient into three groups: mapped unique reads (indicated by red, multiple mapped reads (indicated by blue), and unmapped reads (indicated purple]). The y-axis displays the number of reads in millions, allowing for a comparative assessment of read mapping efficiency and specificity across patients.
