# Supplementary figures and images for: Cardiac biopsies reveal differences in transcriptomics between left and right ventricle in patients with or without diagnostic signs of heart failure
Source: Sci Rep. 2024 Mar 9;14:5811. doi: 10.1038/s41598-024-56025-1 (PMC10924960; doi:10.1038/s41598-024-56025-1)

Supplementary Figure S1.

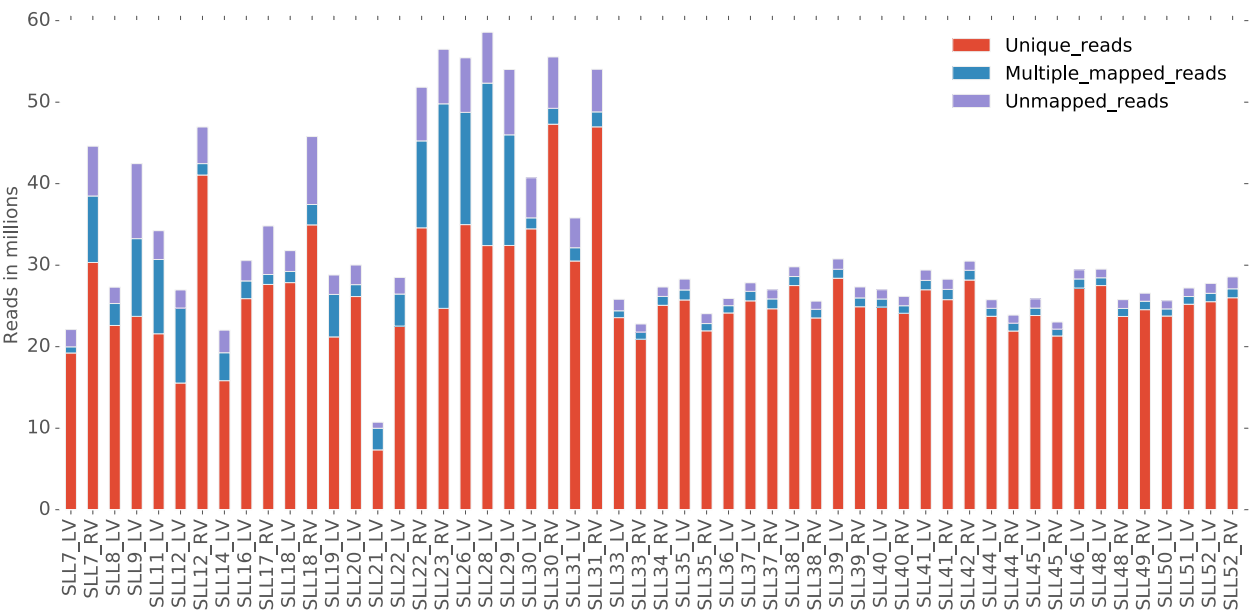

Supplement: Supplementary file 5 — Supplementary Figure S1. [file 41598_2024_56025_MOESM5_ESM.pdf]
